# Pathologic fracture and metastatic spinal cord compression in patients with prostate cancer and bone metastases

**DOI:** 10.1186/1471-2490-10-23

**Published:** 2010-12-22

**Authors:** Carsten Nieder, Ellinor Haukland, Adam Pawinski, Astrid Dalhaug

**Affiliations:** 1 Department of Oncology and Palliative Medicine, Nordland Hospital, Bodø, Norway; 2 Institute of Clinical Medicine, Faculty of Health Sciences, University of Tromsø, Tromsø, Norway

## Abstract

**Background:**

The purpose of this study was to determine the risk factors for and incidence as well as prognostic impact of pathologic fracture (PF) and metastatic spinal cord compression (MSCC) in patients with bone metastases (BM) from prostate cancer.

**Methods:**

Retrospective cohort study including 61 consecutive patients seen at Nordland hospital's department of oncology between 2007 and 2009. The initial diagnosis of BM might have been made earlier. Twenty-nine patients (48%) received taxotere and 72% zoledronic acid after diagnosis of BM.

**Results:**

Median actuarial survival after diagnosis of BM was 23 months. Six patients (10%) were alive at 5 years. Bone pain at baseline was present in 49% of patients. Eighty-nine percent required external beam radiotherapy and/or radioisotopes after diagnosis of BM. Seventeen patients (28%) developed at least one major skeletal complication, i.e. MSCC or PF (4 of them developed more than one). The actuarial risk was 44% at 4 and 5 years. Most events developed before treatment with zoledronic acid and/or taxotere. Median survival from diagnosis of either MSCC or PF was 11 months (5 months from MSCC). We did not identify statistically significant risk factors for development of major skeletal complications. Serum alkaline phosphatase above median value and age less than or equal to 70 years were the only risk factors approaching significance.

**Conclusions:**

We found high rates of major skeletal complications in this unselected contemporary group of patients. Identification of risk factors might guide the development of early interventions aiming at prevention of MSCC and PF.

## Background

Bone metastasis is a common complication in patients with advanced stage prostate cancer and might even be found already at first clinical diagnosis [1,2]. Increasing extent of spread might compromise bone stability, resulting in pathologic facture (PF) [3]. In addition, at least 5-10% of patients might develop metastatic spinal cord compression (MSCC) with or without vertebral fracture [4]. Prognosis after onset of MSCC is limited. Factors predicting for these two major skeletal complications in patients managed by practitioners outside of prospective clinical trials are not completely understood. To study the incidence, outcome and risk factors for PF and MSCC in men with prostate cancer and skeletal metastases, a retrospective cohort study was performed.

## Methods

A retrospective analysis, which included all patients with prostate cancer and bone metastases treated at the authors' institution during 2007, 2008 and early 2009 was performed. The authors' institution is a community hospital in rural Norway, which is the only oncology care provider and services the complete population of the county, i.e. approximately 236,000 inhabitants. Thus, the 61 consecutive patients included in this study represent an unselected population. Follow-up information was available in all patients. The initial diagnosis of bone metastases might have been made before 2007. Our laboratory provided the following information on normal ranges: haemoglobin (Hb) 13.4-17.0 g/dL, serum alkaline phosphatase (ALP) <105 U/L, serum lactate dehydrogenase (LDH) <205 U/L and serum calcium 2.15-2.55 mmol/L. We used the Kaplan-Meier method to generate actuarial survival curves. Patients without event were censored at last clinical follow-up. Survival was calculated from the date of imaging diagnosis of bone metastases (typically by isotope bone scan) or from development of other analysed events. Survival curves were compared with the log rank test. Afterwards, statistically significant prognostic factors for survival were examined by multivariate Cox regression analysis (forward stepwise data selection method). Wilcoxon- and Kruskal-Wallis-tests were used to compare the baseline characteristics between different groups. Risk factors for development of PF and MSCC were evaluated with the Fisher exact test and confirmed by actuarial Kaplan-Meier analyses as these were time-dependent events. PF was defined as a bone broken, not by trauma alone, but so weakened by metastatic spread as to break with abnormal ease [5,6]. MSCC was defined as compression by direct pressure and/or induction of vertebral collapse or instability by metastatic spread that threatens or causes neurological disability [5,6]. Vertebral deformities (consistent with fracture) were identified by direct visualisation using the Genant semi-quantitative grading scale [7,8]. Plain radiography was used except for some patients who had computed tomography scans only. Synchronous bone metastasis was defined as simultaneous diagnosis of prostate cancer and metastatic spread to the bones. A p-value ≤ 0.05 was considered statistically significant. No correction for multiple testing was performed. The study was performed as a retrospective analysis of skeletal complications. As a quality of care analysis where data was fully de-identified, no approval from the Regional Committees for Medical and Health Research Ethics (REK) was necessary.

## Results

The patient characteristics and length of follow-up are shown in Table 1. Treatment consisted of different types of androgen suppression regimens incl. steroids and palliative external beam radiotherapy in patients with bone pain, MSCC or surgically stabilized PF. Administration of other treatments is also shown in Table 1. Twenty-nine patients (48%) received taxotere after diagnosis of bone metastases and 7 of these also proceeded to second-line treatment with mitoxantrone. The initial number of bone metastases on radioisotope bone scan was significantly higher in patients with synchronous presentation compared to metachronous presentation, p = 0.05. Patients with synchronous presentation also had significantly higher median prostate-specific antigen (PSA) value, p < 0.01, ALP value (p < 0.01) and LDH value (p = 0.03), as shown in Table 1. No other significant differences in baseline characteristics were found between these two groups, which did not differ significantly in overall survival or development of MSCC and PF.

**Table 1:** Patient characteristics

Parameter	All 61 patients	23 patients with bone metastases at first cancer diagnosis	38 patients with metachronous diagnosis of bone metastases
Median age, range (years)*	69, 56-86	67, 57-79	73, 56-86
Median age at first cancer diagnosis, range (years)	68, 53-80	67, 57-79	68, 53-80
Median ECOG PS, range*	1, 0-2	1, 0-2	1, 0-2
Median interval, range (months)	22, 0-159	0	51, 5-159
Median PSA doubling time, range (months)*	Not applicable	Not applicable	2.5, 1-8
Median PSA, range (μg/L)*	49, 3.9-10,302	173, 12-10,302	23, 3.9-727
Median Hb, range (g/dL)*	13.7, 10.0-16.8	13.9, 10.0-16.8	13.4, 10.2-15.2
Median ALP, range (U/L)*	118, 44-2742	184, 56-2742	102, 44-641
Median LDH, range (U/L)*	206, 158-784	357, 190-784	196, 158-781
Hypo- or hypercalcemia*	0	0	0
Bone pain*	30	11	19
	49%	48%	50%
Gleason score < 7, 7, >7**	6, 11, 27	2, 5, 11	4, 6, 16
	14%, 25%, 61%	11%, 28%, 61%	15%, 23%, 62%
Other distant metastases*	19	8	11
	31%	35%	29%
≤10 bone metastases, >10,	26, 29, 6	6, 12, 5	20, 17, 1
superscan	43%, 48%, 10%	26%, 52%, 22%	53%, 45%, 3%
Initial prostatectomy or radical	10	0	10
radiotherapy	16%		26%
Taxotere treatment	29	12	17
	48%	52%	45%
Zoledronic acid treatment	44	18	26
	72%	78%	68%
Radioisotope treatment	8	5	3
	13%	22%	8%
Alive at the time of analysis	34	14	20
	56%	61%	53%
Median follow-up of living patients, range (months)	25, 6-84	29, 9-84	23, 6-67

* when diagnosed with bone metastases

** unknown in 5 patients with synchronous and 12 patients with metachronous diagnosis

ECOG PS: Eastern Cooperative Oncology Group performance status, PSA: prostate-specific antigen, Hb: haemoglobin, ALP: alkaline phosphatase, LDH: lactate dehydrogenase

Median actuarial survival after diagnosis of bone metastases was 23 months. Six patients (10%) were alive at 5 years. The parameters shown in Table 2 were examined for their prognostic impact by comparing actuarial survival curves (log rank test). While Hb at the time of bone metastases detection was not significant when using the median value as cut-off, a significant association between Hb ≤ 12.0 g/dL and short survival was found, p = 0.03. The 2-year survival rate in patients with Hb ≤ 12.0 g/dL was 25%. Performance status also was significant, p = 0.01. Patients with Eastern Cooperative Oncology Group (ECOG) performance status of 2 had 33% 2-year survival, those with ECOG 1 43% and those with ECOG 0 77%. In these analyses, age > 70 years, ALP above median value, and LDH above median value also predicted for significantly shorter survival, p ≤ 0.05. A multivariate analysis confirmed performance status, ALP and LDH as independent prognostic factors for survival, p ≤ 0.05. Chemotherapy-treated patients had a 2-year survival rate of 65%. Patients managed without chemotherapy because of age, poor performance status and other contraindications had a 2-year survival rate of 39% (median survival 29 months with chemotherapy versus 17 months without).

**Table 2:** Prognostic factors for survival in all 61 patients

Parameter	Unfavourable prognosis (comparison of actuarial survival curves, log rank test)	Multivariate analysis (Cox proportional hazards model)
Median age (years)*	age > 70 years, p = 0.04	Not significant
Median age at first cancer diagnosis (years)	Not significant	
Median ECOG PS*	Increasing ECOG PS, p = 0.01	Significant, p = 0.03
Median interval (months)**	Not significant	
Median PSA doubling time (months)**	PSA doubling time < 3 months, p = 0.03	Not significant
Median PSA (μg/L)*	Not significant	
Hb (g/dL)*	Hb ≤ 12.0 g/dL, p = 0.03	Not significant
Median ALP (U/L)*	ALP > 118 U/L, p < 0.01	Significant, p = 0.02
Median LDH (U/L)*	LDH > 206 U/L, p < 0.01	Significant, p = 0.03
Hypo- or hypercalcemia*	Not significant	
Bone pain*	Not significant	
Gleason score	Not significant	
Other distant metastases*	Not significant	
≤10 bone metastases, >10, superscan	Not significant	

ECOG PS: Eastern Cooperative Oncology Group performance status, PSA: prostate-specific antigen, Hb: haemoglobin, ALP: alkaline phosphatase, LDH: lactate dehydrogenase

* when diagnosed with bone metastases

**only available in patients with metachronous diagnosis

**Figure 1:**
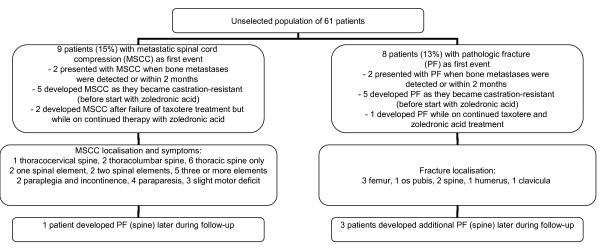
**Overview on development of major skeletal complications in 61 contemporary patients with bone metastases from prostate cancer**.

**Figure 2:**
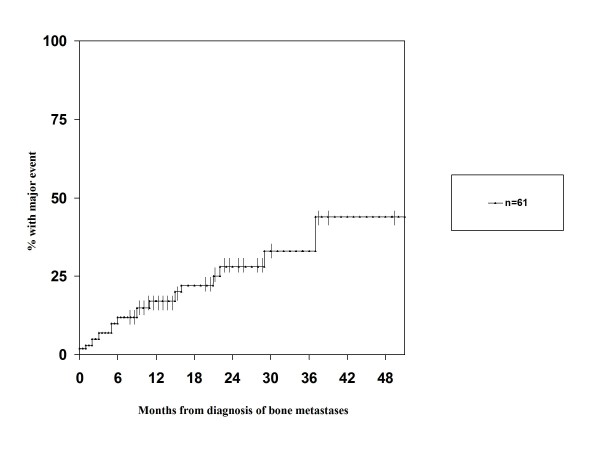
**Kaplan-Meier estimate of time to development of first major skeletal complication (metastatic spinal cord compression and pathologic fracture) in 61 patients with bone metastases from prostate cancer**.

Eighty-nine percent of patients required external beam radiotherapy and/or radioisotopes for bone metastases treatment during follow-up. None of the patients had disorders of calcium metabolism at diagnosis. No episodes of hypercalcemia developed during follow-up. Seventeen patients (28%) developed at least one major skeletal complication, i.e. MSCC or PF (4 of them developed more than one). As shown in Figure 1, 9 patients suffered from MSCC and 8 from PF as first major complication. Most events developed before treatment with zoledronic acid and/or taxotere. In addition, 2 patients were diagnosed with osteoporotic vertebral fractures that were treated conservatively. These patients are not included in the following actuarial analyses and exploration of risk factors for major skeletal complications. Median survival from diagnosis of either MSCC or PF was 11 months, range 1-84. Median survival from diagnosis of MSCC was 5 months, range 1-63. Figure 2 shows the time to development of the first major skeletal complication. In this actuarial analysis, 44% of patients had developed at least one major skeletal complication at 4 and 5 years. We identified only two potential risk factors for development of major skeletal complications, serum ALP above median value (p = 0.1) and age ≤ 70 years (p = 0.2). However, with only 17 patients no statistical significance was obtained.

## Discussion

The present study is to our best knowledge the only community-based contemporary series examining the incidence, outcome and risk factors for development of major skeletal complications in patients treated for bone metastases from prostate cancer. As the group size and thus statistical power is limited, confirmatory studies in larger databases should be attempted. Our data are derived from a representative unselected patient population, actually including all men with bone metastases from prostate cancer in a well defined geographical region. Data from the Cancer Registry of Norway http://www.kreftregisteret.no have shown that the age-adjusted prostate cancer incidence rate per 100,000 person years in Norway (96.6) is comparable to that in our region (100.2) [9]. In Norway, treatment recommendations are based on national guidelines. Therefore, we have no reason to believe that our data are flawed by serious selection bias. Nevertheless, retrospective analyses should be interpreted cautiously. Prospective evaluation might provide a better picture of skeletal complications in patients with metastatic prostate cancer.

Treatment was individualised, taking into account age, organ function, performance status, symptoms, life expectancy etc. Forty-eight percent of patients received taxotere after diagnosis of bone metastases and some patients also had second-line treatment with mitoxantrone. The 2-year survival rate was 65% in chemotherapy-treated patients and 39% in others. This difference is only partially attributable to treatment as this was a retrospective study where several selection factors influenced the choice of treatment. Median overall survival was 23 months. Established prognostic models such as the one developed by Halabi et al., which includes, e.g., performance status, Hb, ALP, LDH and PSA, were derived from large databases [10]. With the current number of patients, only the strongest prognostic factors for survival could be identified. However, these results are in line with the ones published by Halabi et al. [10].

Forty-nine percent of patients had bone pain already at baseline and 89% eventually required external beam radiotherapy and/or radioisotopes during the course of disease. In a recent Japanese study, the corresponding figures were 45% and 51%, respectively [11]. In that study, 14% of patients experienced bone fracture compared to 13% in the present one. A study from the US arrived at a rate of radiotherapy utilisation of 89% and development of PF in 23% [12]. The threshold for radiotherapy utilisation versus medical management of pain might vary from case to case or centre to centre. This introduces a possible source of bias when reporting skeletal-related events (SRE) and was the reason for focusing the present study on major skeletal complications. It should be noticed that androgen deprivation therapy might result in bone loss and osteoporotic fractures [13]. Depending on imaging modality, differentiation between osteoporotic and metastatic fractures might be challenging. In our own series, only 2 patients were thought to have had osteoporotic fractures. When adding these patients to the 9 with PF, the overall bone fracture rate increased to 18%. Additional PF after the first event developed in 3 patients. Most events occurred before initiation of treatment with zoledronic acid and/or taxotere, i.e. during the phase where patients developed castration-resistant disease. In some cases, PF led to the diagnosis of prostate cancer. Berruti et al. reported that 5% of all SRE developed before the onset of castration-resistant disease [14]. Not all patients in our series were treated with zoledronic acid. The main reasons for withholding treatment were severe renal dysfunction and anticipated short survival, e.g., in patients with considerable extraskeletal metastases unfit for chemotherapy. Zoledronic acid significantly reduces the skeletal morbidity rate [15]. Early treatment initiation in patients at high risk of PF and other SRE might thus be beneficial. We will later discuss the potential risk factors for SRE, which includes a broader range of events, and major skeletal complications, which we defined as MSCC and PF. In a series of 68 surgically treated patients with PF or impending fracture, median survival from surgery was 1 year [16]. In our own series, all 8 patients with PF survived for more than 1 year (median 16 months).

MSCC developed in 15% of our patients. This rate is slightly higher than in other studies. However, the definition of MSCC or impending MSCC is somewhat subjective and varies from centre to centre. We did not screen for MSCC in asymptomatic patients. Bayley et al. reported that occult subarachnoid space or spinal cord compression was diagnosed in 32% of patients examined with magnetic resonance imaging [17]. Median survival from diagnosis of MSCC was comparable to other series [4]. As with PF, most MSCC events occurred before initiation of treatment with zoledronic acid and/or taxotere. Hypercalcemia, which was not observed in this series, is a relatively rare event in prostate cancer patients (8% in the study by Tucci et al. [18]). However, these patients appear to have higher LDH values and bone pain as well as a poor prognosis and higher risk to develop SRE. Another important SRE risk factor is ALP level, in this case bone ALP [3]. Baseline cross-linked N-telopeptides of type I collagen (NTx) was also identified as predictor of SRE [19]. That study was performed in patients treated with zoledronic acid. A different study found that SRE risk can be predicted by disease extent in bone and pain score [14]. Our own study was hampered by the limited number of events. Even a broader definition of SRE would have resulted in low statistical power. It is nevertheless noticeable that serum ALP level approached statistical significance. More specific markers of bone turnover were not measured in our patients. Presence of bone pain at baseline and extent of spread on isotope bone scans was not associated with increased risk. In the study by Smith et al., which looked at bone ALP, this parameter was the most important risk factor [3].

## Conclusions

Bone pain, MSCC and PF continue to be common events during the course of prostate cancer with skeletal metastases. Serious SRE contribute significantly to morbidity and pose challenges to those involved in palliative care for these patients. Prevention of skeletal morbidity is a worthwhile goal in high-risk patients. Candidate risk factors defining high-risk groups, e.g., ALP, NTx and hypercalcemia, need to be studied in larger databases. Future studies might need to analyse patients already receiving zoledronic acid and/or taxotere separately from those continuing on endocrine treatment alone.

## Competing interests

The authors declare that they have no competing interests.

## Authors' contributions

CN, EH and AD participated in the design of the study, EH, AD and AP collected patient data and follow-up information, CN carried out the statistical analysis, CN and AP drafted the manuscript. All authors read and approved the final manuscript.

## Pre-publication history

The pre-publication history for this paper can be accessed here:

http://www.biomedcentral.com/1471-2490/10/23/prepub
